# A Health Impact Assessment of Proposed Public Transportation Service Cuts and Fare Increases in Boston, Massachusetts (U.S.A.)

**DOI:** 10.3390/ijerph110808010

**Published:** 2014-08-07

**Authors:** Peter James, Kate Ito, Jonathan J. Buonocore, Jonathan I. Levy, Mariana C. Arcaya

**Affiliations:** 1 Departments of Epidemiology and Environmental Health, Harvard School of Public Health, 401 Park Drive, Boston, MA 02215, USA; 2 Metropolitan Area Planning Council, 60 Temple Place, Boston, MA 02111, USA; E-Mails: kito@mapc.org (K.I.); marcaya@mapc.org (M.C.A.); 3 Harvard Center for Health and the Global Environment, 401 Park Drive, Boston, MA 02215, USA; E-Mail: jjb194@mail.harvard.edu; 4 Boston University School of Public Health, 715 Albany St. Talbot Building, Boston, MA 02118, USA; E-Mail: jonlevy@bu.edu; 5 Harvard Center for Population and Development Studies, 9 Bow Street, Cambridge, MA 02138, USA; E-Mail: mca767@mail.harvard.edu

**Keywords:** Health Impact Assessment, public transportation, air pollution, physical activity, crashes, monetization

## Abstract

Transportation decisions have health consequences that are often not incorporated into policy-making processes. Health Impact Assessment (HIA) is a process that can be used to evaluate health effects of transportation policy. We present a rapid HIA, conducted over eight weeks, evaluating health and economic effects of proposed fare increases and service cuts to Boston, Massachusetts’ public transportation system. We used transportation modeling in concert with tools allowing for quantification and monetization of multiple pathways. We estimated health and economic costs of proposed public transportation system changes to be hundreds of millions of dollars per year, exceeding the budget gap the public transportation authority was required to close. Significant health pathways included crashes, air pollution, and physical activity. The HIA enabled stakeholders to advocate for more modest fare increases and service cuts, which were eventually adopted by decision makers. This HIA was among the first to quantify and monetize multiple pathways linking transportation decisions with health and economic outcomes, using approaches that could be applied in different settings. Including health costs in transportation decisions can lead to policy choices with both economic and public health benefits.

## 1. Introduction

Public transportation ridership in the United States (US) is at its highest level in 57 years, with 10.7 billion trips taken on public transportation in 2013 [1]. Transportation systems help shape communities and affect safety, physical activity, healthcare access, and the environment [2]. Traditionally driven by budgetary considerations, public transportation policy rarely incorporates information on downstream health effects. Mounting evidence demonstrates that transportation decisions have health consequences [3], many of which have economic impacts for both individuals and governments. Estimating health consequences of transportation policy decisions is challenging, especially within a decision-relevant timeline, but such information would provide policymakers and the public with important insight into health impacts as well as their attendant costs.

Health Impact Assessment (HIA) is a process that uses an array of data sources and analytic methods to help decision-makers understand the health implications of a proposed project, plan, or policy [4]. A recent analysis of HIAs conducted in the US between 2005 and 2012 found that 21 (25.9%) focused on transportation [5]. Of these, 15 examined environmental endpoints (almost exclusively air quality), and nine utilized quantitative modeling. Three HIAs specifically concentrated on public transportation. These HIAs primarily used literature review, consultation with experts, and modeling to review the expansion of a public transportation line, synthesize evidence on proposed cuts in public transportation funding, and complement an environmental impact statement. The HIA analyzing cuts to public transportation funding found that public transportation could benefit health by reducing air pollution, increasing physical activity, improving mental health, and enhancing community social capital. However, none of the HIAs examining transportation issues resulted in modifications to the decision being assessed, potentially because the HIAs did not provide the requisite information to inform a pending decision. The use of HIA to examine transportation decisions has expanded in recent years. The Health Impact Project, a database of HIAs across the United States, shows that there are currently over 60 transportation-related HIAs completed or in progress [6]. At the time of this study, Massachusetts was in the process of piloting a transportation HIA as a part of the state’s Healthy Transportation Compact, which requires the implementation of HIAs for use by planners and transportation administrators [7].

**Figure 1:**
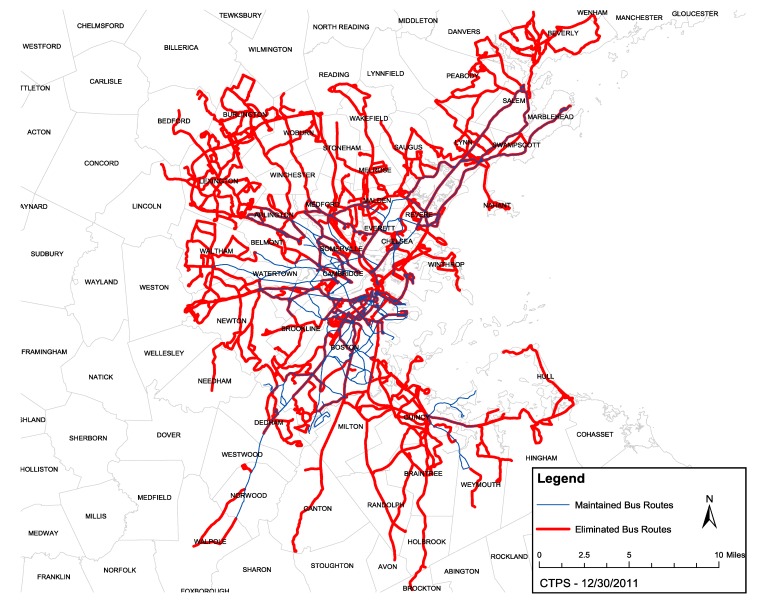
Eliminated bus routes under Scenario 2 (Image from CTPS 2011 [8]).

We present an HIA on public transportation funding decisions in the Boston, Massachusetts region as an example of effective use of HIA to influence transportation policy. The Massachusetts Bay Transportation Authority (MBTA) faced a projected budget deficit for fiscal year 2013 of $161 million. The MBTA was obligated to close this gap, but had limited means to raise revenue or reduce costs. Working under these fiscal and political constraints, in January 2012 the MBTA proposed two scenarios of fare increases and service cuts aimed at closing its projected deficit. Fares would have increased by 43% and service reductions would have affected 34–48 million trips each year under Scenario 1. Under Scenario 2, fares would have increased by 35% and service reductions would have affected 53–64 million trips each year, including significant elimination of regional bus routes (Figure 1).

Contentious debate surrounded the MBTA fare increases and service cuts; however, health was not a focus of the conversation. Boston’s regional planning agency, the Metropolitan Area Planning Council (MAPC), sought to inform decision-makers on the health impacts of the MBTA funding decision. Working with colleagues from the Harvard School of Public Health and the Boston University School of Public Health, MAPC conducted an HIA to estimate the comprehensive health and economic effects of the proposed MBTA fare increases and service cuts. This work was independently funded by MAPC. We considered numerous health pathways, as well as two direct economic impacts. The primary audience for the HIA was the Massachusetts Legislature, so we considered the overarching evidence of how the MBTA serves as a fundamental health resource for the region.

## 2. Experimental Section

### 2.1. HIA Process

The standard steps of HIA include selecting appropriate projects during a screening phase, outlining pathways through which the project could affect health through a scoping phase, developing predictions of expected health effects due to the project during an assessment phase, creating recommendations to optimize health or mitigate negative health effects through a recommendation phase, writing up the HIA and communicating results during a reporting phase, and tracking the impact of the HIA in a monitoring phase [9]. The MBTA HIA was a rapid HIA completed in about eight weeks to provide information during public hearings on the MBTA funding scenarios.

### 2.2. HIA Assessment

**Figure 2:**
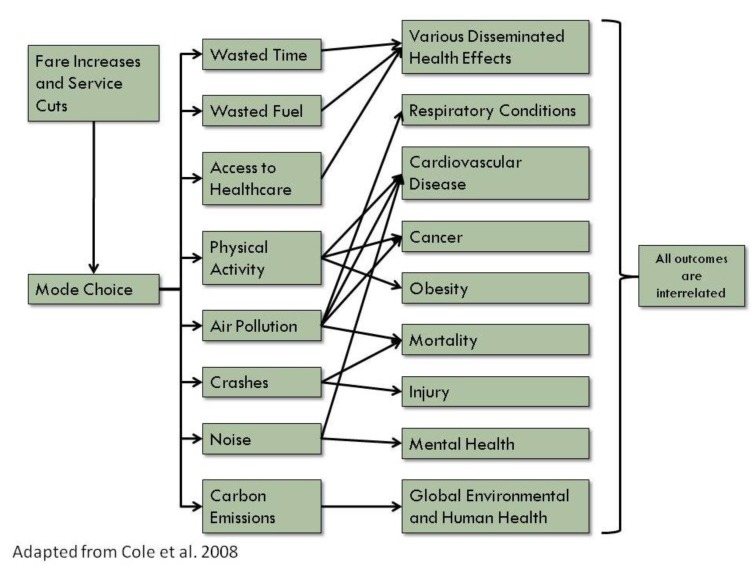
Pathways for the Health Impacts of Public Transportation.

Because stakeholder interest indicated the value of an HIA focused on MBTA service cuts and fare increases, the project was rapidly screened in. During scoping, we utilized previously-developed conceptual models for transportation HIAs [10], but focused on environmental, social, and economic pathways that could be quantitatively analyzed (Figure 2). 

Data allowed us to quantify the following pathways: 1. Time Spent in Traffic and Fuel Costs; 2. Air Pollution; 3. Physical Activity; 4. Crashes; 5. Access to Healthcare; 6. Carbon Dioxide Emissions; 7. Noise. We note that all pathways have plausible connections to health outcomes, although the first pathway only includes direct economic consequences, and the monetization of carbon emissions encompasses many pathways, as described in detail below. While this HIA focuses on outcomes that can be quantified, we identified many pathways during scoping that are primarily qualitative, such as social equity and mental health. Because of our short time frame and because the HIA was intended to inform a budget debate, we focused on quantitative outcomes; however we emphasize that the absence of numbers does not make such topics less important.

Most pathway analyses estimated health and economic impacts as a function of commuting mode shifts, motor vehicle miles travelled, time spent driving, and vehicle emissions. We collaborated with the Central Transportation Planning Staff (CTPS) of Boston’s Metropolitan Planning Organization to obtain these data. Additionally, we informed the state legislature and the MBTA that we were conducting this HIA. We developed estimates of both health and indirect economic impacts of each scenario to the population of the region using CTPS data, and leveraging collective expertise in air quality, environmental health, and physical activity.

#### 2.2.1. MBTA Service Area

The MBTA serves 175 cities and towns in a 3244 square mile area [11]. Based on the 2010 Census, the population reached by the MBTA is over 4.8 million, and ridership on a typical weekday is approximately 1.3 million trips. For the MBTA service area, the population-weighted mean percent below poverty at the municipal level is 10% (range 1%–26%), while the median household income at the municipal level ranged from $32,000 to $165,000 [12]. Over a quarter of the population in the MBTA service area lives in a community with a median household income below $51,000.

#### 2.2.2. Time Spent in Traffic and Fuel Costs

CTPS models indicated that fare increases and service cuts would lower public transportation ridership, increasing the numbers of drivers on the region’s roads and consequently increasing the number of hours residents spend in cars.

To estimate the cost of increased time spent in traffic under the proposed scenarios, we compared conditions under full MBTA service to vehicle hours traveled (VHT) and vehicle miles traveled (VMT) projected for the two proposed scenarios [8]. Vehicle occupancy and monetary value of time were taken from the Texas Transportation Institute Urban Mobility Report [12].

Average traffic speed across the region was calculated for baseline conditions and each scenario by dividing VMT by VHT and was used to estimate time in traffic and fuel consumption. Fuel economy in gallons per mile for trucks and automobiles was estimated separately [12]. We calculated VMT for automobiles and trucks using the regional personal/commercial vehicle mix (95% automobiles, 5% trucks) and multiplied the VMT for automobiles and trucks by fuel economy for each vehicle type to estimate fuel consumption (see equations below). The fuel consumed was then multiplied by the cost of fuel for each vehicle type, assuming that automobiles are fueled exclusively by gasoline and trucks are fueled exclusively by diesel, using estimates for Massachusetts 2010 average gasoline cost of $2.86/gallon and an average diesel cost of $3.16/gallon (2012 dollars) [12]. We then took the difference between estimates at baseline and each scenario to provide an estimate of the cost of excess fuel due to congestion related to the MBTA scenarios:

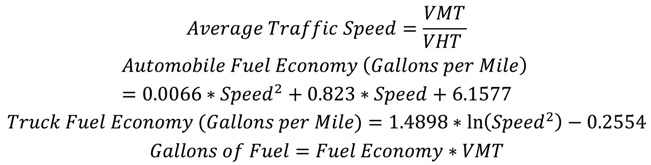


#### 2.2.3. Air Pollution

Extensive epidemiological evidence links air pollution to mortality and hospitalizations due to asthma, lung disease, heart attacks, ischemic heart disease, and cardiovascular disease [13,14,15,16]. We developed estimates of health impacts due to changes in vehicular air emissions by linking an emissions model (MOBILE 6.2) used by the US Environmental Protection Agency (EPA), a reduced-form model linking emissions with county-level concentrations, peer-reviewed literature on the relationship between air pollution and health, and baseline population data.

Changes in county-level concentrations of fine particulate matter (PM_2.5_) air pollution attributable to emissions of PM_2.5_, NO_x_, or SO_2_ under each scenario were estimated using a Source-Receptor Matrix developed for the US EPA [17]. We obtained county-level baseline data on hospitalization rates for asthma, chronic obstructive pulmonary disease (COPD), myocardial infarction (MI), and cardiovascular disease (CVD) from MassCHIP [18], and data on mortality rates from the US Centers for Disease Control and Prevention [19]. Health impacts due to air quality changes were calculated based on concentration-response relationships compiled in the EPA Environmental Benefits Mapping and Analysis Program (BenMAP) [20]—mortality [14,21], hospitalizations for asthma [15], CVD [13,22], MI [13], and COPD [23].

#### 2.2.4. Physical Activity

In the counties served by the MBTA, approximately 19% of adults are obese and 16% report no daily physical activity [24]. Commuting patterns influence daily physical activity. Although Americans only walk about six minutes daily, public transportation users walk a median of 19 min daily [25]. Estimates show that individuals walk an additional 8.3 min per day when they switch from driving to public transportation [26].

To calculate the mortality and economic impact associated with decreased walking, we used the web-based Health Economic Assessment Tool (HEAT) from the World Health Organization (WHO) [27]. HEAT was developed with the guidance of an advisory group of international experts in health, epidemiology, health economics, transport economics, practice/advocacy, and policy development and implementation.

CTPS estimated the number of individuals switching from public transportation to driving under the two scenarios. Physical activity, calories expended, and obesity risk estimates came from National Household Travel Survey data [26]. Using HEAT, we simulated an intervention in the population that shifted from public transportation to driving where participants decreased walking by an average of 8 min per weekday, or an average of 40 min per week per person. We conservatively estimated that those commuting by public transportation were already getting 30 min of walking per day, or 150 min per week. We then input baseline rates for mortality from MassCHIP data [18] into HEAT, which used dose-response functions to estimate the increase in mortality due to the decreased walking in this population.

#### 2.2.5. Crashes

Decreasing public transportation service can increase traffic-related injury risk by shifting a portion of daily trips from a safer mode of travel (e.g., bus or train) to a more dangerous mode (e.g., automobile travel). According to national transportation statistics, the risk of fatal injury per person-trip by bus in the U.S. is 1/23 as much as by car and the risk of non-fatal injury is 1/5 as much for bus trips compared to automobile trips [28]. In light of differing risk by transportation mode, we estimated how decreases in public transportation use could increase time spent in automobiles and subsequently affect crash fatalities.

Traffic fatality rates per VMT were taken from 2009 National Highway Traffic Safety Administration estimates for the state of Massachusetts (0.61 fatalities per 100 million VMT, 2009) [29]. We estimated expected traffic fatalities by multiplying projected increases in VMT under the two proposed scenarios by the fatality rate per VMT for Massachusetts. To estimate the costs of congestion and crashes, we multiplied the projected increases in VMT by the cost per VMT of congestion in large urban areas from the American Automobile Association (AAA) [30].

#### 2.2.6. Access to Healthcare

Access to transportation is a requirement for access to healthcare. Individuals who cannot easily reach healthcare facilities visit their doctor less frequently for regular checkups, as well as for serious illness, affecting health outcomes and healthcare costs [31]. For carless households, MBTA service is fundamental to reach preventative healthcare resources in an affordable and reliable way. We quantified how many carless households live in neighborhoods that were both facing MBTA service losses and had no essential healthcare resources within walking distance.

To estimate how the proposed scenarios would affect the ability of public transportation-dependent households to access healthcare, we mapped healthcare locations, routes that would be affected by service cuts, and Census data on households without cars. We identified areas that offered public transportation access to healthcare facilities, but would have been isolated from public transportation access to care if proposed cuts went into effect. To calculate access to healthcare, we used the *infoUSA* data on healthcare locations, Census 2010 data, and the 2006–2010 American Community Survey (ACS). The maintained and eliminated MBTA service routes for the two scenarios were mapped using Geographic Information Systems (GIS). Using a half-mile radius, which is a commonly accepted upper bound of what Americans are willing to walk to reach destinations [32,33,34], we created a buffer around the eliminated routes in ArcGIS, defining this as the area affected by potential service losses. We then identified Census blocks that would have lost public transportation access to healthcare facilities and have no healthcare facilities within walking distance. We obtained household counts for these affected neighborhoods and then used tract-level ACS data to estimate the proportion of the affected population likely to be carless in each neighborhood. Finally, we applied neighborhood-level car access rates to each affected block’s population to estimate the carless population that would lose public transportation access to healthcare. We recognize that there are numerous complex factors that influence healthcare access and utilization (e.g., access to providers that accept a specific insurance policy) that we were unable to incorporate into our assessment; however, we examined geographic access to healthcare because studies have shown that geographic proximity affects healthcare utilization [35] and decreasing transportation options to healthcare facilities would serve as a barrier to healthcare utilization.

#### 2.2.7. Carbon Dioxide Emissions

Both scenarios would have increased carbon dioxide emissions due to greater personal automobile use, congestion, and wasted fuel, contributing to global climate change and its subsequent health effects [36,37,38]. To estimate the cost of carbon, we used social cost of carbon estimates from the National Academy of Sciences (NAS) [39]. We took a midrange estimate of the social cost of carbon ($31.18 2012 USD per ton) and multiplied it by the annual increased carbon emissions from the CTPS transportation models. This estimate was based on the social cost of carbon at the time of the initial issue; the social cost of carbon has been updated since then.

#### 2.2.8. Noise

In Boston, approximately 16% of the population lives within 100 m of a 4-or-more lane highway, where vehicle travel is likely to cause noise disturbances [40]. Exposure to excessive noise may induce hearing loss and negatively impact mental and cardiovascular health [41]. This analysis focused on the change in number of individuals exposed to noise levels greater than 60 decibels (dBA) under the scenarios because transportation noise levels above 60 dBA have been associated with hypertension [42,43].

We used a look-up table derived from Version 2.5 of FHWA’s Transportation Noise Model (TNM) [44] to associate vehicle volume and speed with noise levels in each traffic analysis zone (TAZ), linearly interpolating between reported values in the look-up tables. We estimated average traffic volume per TAZ by dividing VMT by road length in the TAZ and then applied the TNM to that estimated volume. We focused on individuals living 100 m from major roadways, conservatively applying estimates from a 100 m distance to all individuals within that zone. For this rapid assessment, we estimated the number of exposed individuals by assuming that each TAZ housed 16% of its population within 100 m of a major road, reflecting the proportion of the statewide population living within 100 m of a major road.

#### 2.2.9. Economic Value of Health Endpoints

The value of a statistical life of $8.32 million in 2012 USD was used to monetize mortality endpoints [45]. The values of a hospitalization event were obtained from BenMAP [20]. The total value to society of an individual’s avoidance of a hospital admission has two components: the cost of illness (COI) to society, which includes the total medical costs plus the value of the lost productivity; as well as the willingness to pay (WTP) of the individual, as well as that of others, to avoid the pain and suffering resulting from the illness. BenMAP does not contain WTP estimates for avoided hospital admissions, and therefore estimates of total COI are conservative estimates.

### 2.3. Review Approach

The draft report was produced in collaboration with faculty and students at the Harvard School of Public Health, Harvard Medical School, and Boston University School of Public Health. Early drafts were provided to CTPS, the Massachusetts Department of Transportation, the Massachusetts Department of Public Health, and an independent transportation consultant.

## 3. Results and Discussion

Our HIA projected that fare increases and service cuts to public transportation in the Boston region would have resulted in lost time as more residents sit in traffic; worse air quality; lower levels of physical activity; additional crashes; isolation from basic healthcare resources for hundreds of carless households; increased exposure to high noise levels; and additional greenhouse gas emissions. These estimates were based on the approximate 30,000–49,000 people shifting from public transportation to driving and the fact that current drivers collectively would have spent an additional 18,500–25,100 h per year driving under the proposed scenarios. We found that Scenario 1 would have resulted in approximately 70 new cases of obesity, 10 avoidable deaths, and various morbidity outcomes per year; while Scenario 2 would have produced approximately 120 new cases of obesity and 15 avoidable deaths per year (Table 1).

**Table 1:** Annual impacts of proposed public transportation fare increases and service cuts.

Pathway	Scenario 1: Fares increase by 43%, Service reductions affecting 34–48 million trips each year	Scenario 2: Fares increase by 35%, Service reductions affecting 53–64 million trips each year
Time Spent in Traffic and Fuel Costs	30,400 people shift from public transportation to driving 18,565 additional person-hours spent in traffic for current drivers 7.4 million gallons of gasoline and 451,000 gallons of diesel	48,600 people shift from public transportation to driving 25,100 additional person-hours spent in traffic for current drivers 10.4 million gallons of gasoline and 319,000 gallons of diesel
Air Pollution	0.18 additional deaths, 0.17 additional hospitalizations due to asthma, chronic lung disease, heart attacks, ischemic heart disease, and major cardiovascular events per year	0.26 additional deaths, 0.24 additional hospitalizations due to asthma, chronic lung disease, heart attacks, ischemic heart disease, and major cardiovascular events per year
Physical Activity	250,000 fewer min of walking per day 8.2 million fewer calories burned per day 70 new cases of obesity per year 9 additional deaths per year	403,000 fewer min of walking per day 13.1 million fewer calories burned per day 120 new cases of obesity per year 14 additional deaths per year
Crashes	0.79 new deaths per year	1.15 new deaths per year
Access to Healthcare	550 public transportation-dependent households would be isolated from basic healthcare resources	2200 public transportation-dependent households would be isolated from basic healthcare resources
Carbon Emissions	Over 58,000 additional metric tons of CO_2_ emitted per year	Over 52,000 additional metric tons of CO_2_ emitted per year
Noise	500 additional people will be exposed to more than 60 dB of noise on average per day	2000 additional people will be exposed to more than 60 dB of noise on average per day

In addition to direct health impacts, the proposed changes would have isolated 550–2200 public transportation-dependent households from basic healthcare resources. Carbon dioxide emissions due to additional personal automobile use and increased congestion would have increased by 52,000–58,000 metric tons per year.

These health impacts would have affected a broad swath of individuals across the region and beyond, including both MBTA riders and the general population. Current drivers in the region would have had longer commutes, those who switched from taking public transportation to driving would have decreased their physical activity, and air pollution levels would have increased across the entire region and across adjacent states.

After monetizing all quantifiable pathways, we estimated that fare increases and service cuts to the MBTA system would have resulted in costs that exceed the $161 million budget shortfall that the proposed scenarios sought to address (Table 2). The direct economic costs to commuters were comparable in magnitude to the revenue generated by the fare increases and service cuts, and the health-related costs exceeded $100 million per year in both scenarios, largely attributable to car crashes and physical activity reductions. These costs do not take into account indirect economic consequences of higher transportation costs, which may compete directly with the costs of other living necessities. These tradeoffs and their health consequences on health have been detailed in other HIAs [46].

**Table 2:** Summary of health and economic costs under proposed public transportation service cuts and fare increases.

Annual impact	Costs of Scenario 1: Fares increase by 43%, Service reductions affecting 34–48 million trips each year	Costs of Scenario 2: Fares increase by 35%, Service reductions affecting 53–64 million trips each year
Cost of additional time in traffic	$137.5 million	$186.0 million
Cost of additional fuel	$22.7 million	$31.8 million
Cost of additional car crashes, including crashes with bicycles and pedestrians	$33.6 million	$48.8 million
Cost of additional mortality and hospitalizations for asthma, chronic lung disease, heart attacks, heart disease, and major cardiovascular events due to air pollution	$1.5 million	$2.1 million
Cost of lives lost due to decreased physical activity	$74.9 million	$116.5 million
Cost of carbon emissions	$1.9 million	$1.7 million
Total annual cost	$272.1 million	$386.9 million

### Dissemination and Impact Evaluation

Our HIA, including a 20-page report, one-page executive summary, and infographic, was released on 13 March 2012 at the Massachusetts State House, in time for the last public hearing on the proposed scenarios on 14th March. The report identified the MBTA as a health resource and provided a reference for transportation funding advocates and legislators seeking evidence that the proposed changes would carry significant human and financial costs. The HIA was cited at the final public hearing and received over 25 unique press hits, including interviews on the local television news and radio. Additionally, the HIA was recognized by Human Impact Partners 2012 Annual Awards as the “most effective, efficient quantitative analysis” [47] and was cited in the 2013 Healthy People/Healthy Economy Report Card that called for more extensive funding for informative HIAs [48].

In April 2012, the MBTA closed its budget deficit with a third Scenario not previously proposed, which raised fares by 23%—reduced from the proposed 35%–43%—and instituted only modest service cuts. This third Scenario relied on additional sources of revenue from the state to fill the budget deficit.

In the midst of a time-sensitive, controversial transportation policy decision-making process, our HIA provided information to the public and policymakers on the health effects of the proposed changes to MBTA fares and services, as well as their related costs. We leveraged detailed transportation modeling for the two scenarios and connected the outputs with quantitative approaches for multiple pathways linking transportation with health.

Our HIA had multiple unique attributes that contributed to the visibility and utility of the report. To our knowledge, this was the first HIA conducted by a regional planning agency, leveraging relationships with both transportation modelers and Boston-area academic institutions. This provided credibility across all domains of the report, reinforced by the fact that the HIA was independent and self-funded. Having broad domain expertise also facilitated completion of the HIA on a rapid basis, so the findings were timely. Because the HIA was focused on a specific audience (the Massachusetts Legislature) and decision (operations scenarios for the upcoming fiscal year), we were able to tailor the content appropriately. Rather than a lengthy document read only by public health practitioners, the final HIA was 20 pages and was supplemented with a one-pager with an infographic that concisely demonstrated to legislators that the health and economic costs of the fare increases and service cuts exceeded the budget shortfall that the proposed scenarios sought to address. Both the focus and structure of the HIA attracted wide media attention, underscoring the interest in health information connected to everyday activities such as commuting.

This HIA had a number of limitations related in part to its rapid nature. Because we conducted the HIA in a short time frame (eight weeks), we did not hold stakeholder engagement meetings. While there are uncertainties associated with any HIA, we made a number of simplified assumptions given the data available and need for a timely analysis. Where possible, we attempted to make conservative estimates to avoid overstating the benefits, but the magnitude and direction of some key uncertainties is unknown, and the optimal methods for multiple pathways may differ for HIAs with a longer time horizon. We also did not include a discussion on the distribution of health outcomes and did not include a monitoring section, both of which are found in typical HIAs. While we present one assessment approach, HIA practitioners with more time and resources might consider primary data collection, more refined models of exposures and health risks, more detailed geospatial data, additional pathways, and more complete assessment of the distribution of health impacts among susceptible populations. Primary data collection could have provided more informed assumptions to input into our models. For instance, we assumed that carless households rely on public transportation access to access healthcare; however, surveys on how individuals obtain healthcare in the region would provide more applicable data to examine the impact of the service cuts on healthcare access.

Despite these limitations, the MBTA HIA can serve as a model for a rapid quantitative approach to HIA that can be applied to time-sensitive transportation decisions. Having the appropriate team positioned to conduct the analysis is crucial. As a state agency, MAPC was well-situated to collaborate with other agencies to quickly gather necessary health and transportation data. Our cross-disciplinary and cross-sector approach enabled us to incorporate rapid but peer-reviewed and high-quality quantitative approaches that can be replicated in other communities. While the focus on quantitative and monetized outputs may be too narrow for some stakeholders and decisions, the ability to directly compare health and economic consequences with the transportation budget deficit was a key feature of our HIA that increased its visibility and influence.

## 4. Conclusions

In conclusion, the MBTA HIA demonstrated that the proposed public transportation fare increases and service cuts would have resulted in preventable deaths and hospitalizations as well as indirect economic impacts. Beyond a demonstration of the viability of a highly quantitative rapid assessment HIA, our report contributed to an ultimate decision of less severe fare increases and modest service cuts. The quantitative tools that we developed can be applied in different settings to create estimates of health effects and costs for public transportation funding scenarios, facilitating the inclusion of health and economic consequences into transportation policy decisions.
